# Supplementary material for: lute: estimating the cell composition of heterogeneous tissue with varying cell sizes using gene expression
Source: bioRxiv. 2024 Apr 6:2024.04.04.588105. Preprint. [Version 1] doi: 10.1101/2024.04.04.588105 (PMC11014536; doi:10.1101/2024.04.04.588105)
Supplement: 2 [file NIHPP2024.04.04.588105V1-supplement-1.pdf]

bioRxiv preprint doi: https://doi.org/10.1101/2024.04.04.588105; this version posted April 6, 2024. The copyright holder for this preprint (which was not certified by peer review) is the author/funder, who has granted bioRxiv a license to display the preprint in perpetuity. It is made available under aCC-BY 4.0 International license.

Supplemental figures

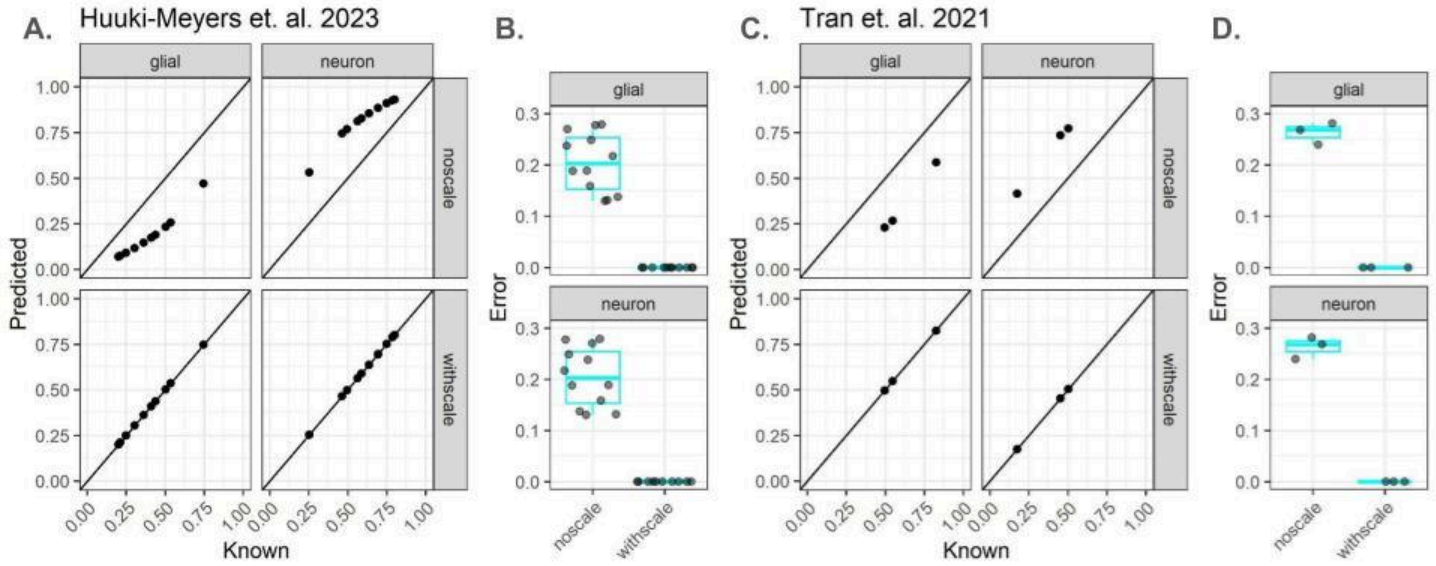

Figure S1 | Pseudobulk simulation results from two DLPFC datasets with *k*=2 cell types. (**A-B**) Pseudobulk results from Huuki-Myers et al. (2023) (46). (**A**) We estimated the cell composition for *k*=2 (neuron and glia) resolution using *NNLS* without (top row) and with (bottom row) scaling for differences in cell sizes, where the known cell composition is on the *x*-axis and the estimated cell composition is on the *y*-axis. The figure is faceted by cell types (neuron and glia) along the columns. (**B**) Boxplots of the absolute error (magnitude difference between the known and predicted cell composition) for the *N*=12 pseudobulk samples, for (top) glia and (bottom) neuron. (**C-D**) Pseudobulk results from Tran et al. (2021) (49). (**C**) We estimated the cell composition using *k*=2 (neuron and glia) using *NNLS* without (top) and with (bottom) scaling for differences in cell sizes where the known cell composition is on the *x*-axis and the estimated cell composition is on the *y*-axis. The figure is faceted by cell types (neuron and glia) along the columns. (**D**) Boxplot of the error (difference between the known and predicted cell composition) for the *N*=3 pseudobulk samples, for (top) glia and (bottom) neuron. Diagonal lines indicate y = x and no error.

bioRxiv preprint doi: https://doi.org/10.1101/2024.04.04.588105; this version posted April 6, 2024. The copyright holder for this preprint (which was not certified by peer review) is the author/funder, who has granted bioRxiv a license to display the preprint in perpetuity. It is made available under aCC-BY 4.0 International license.

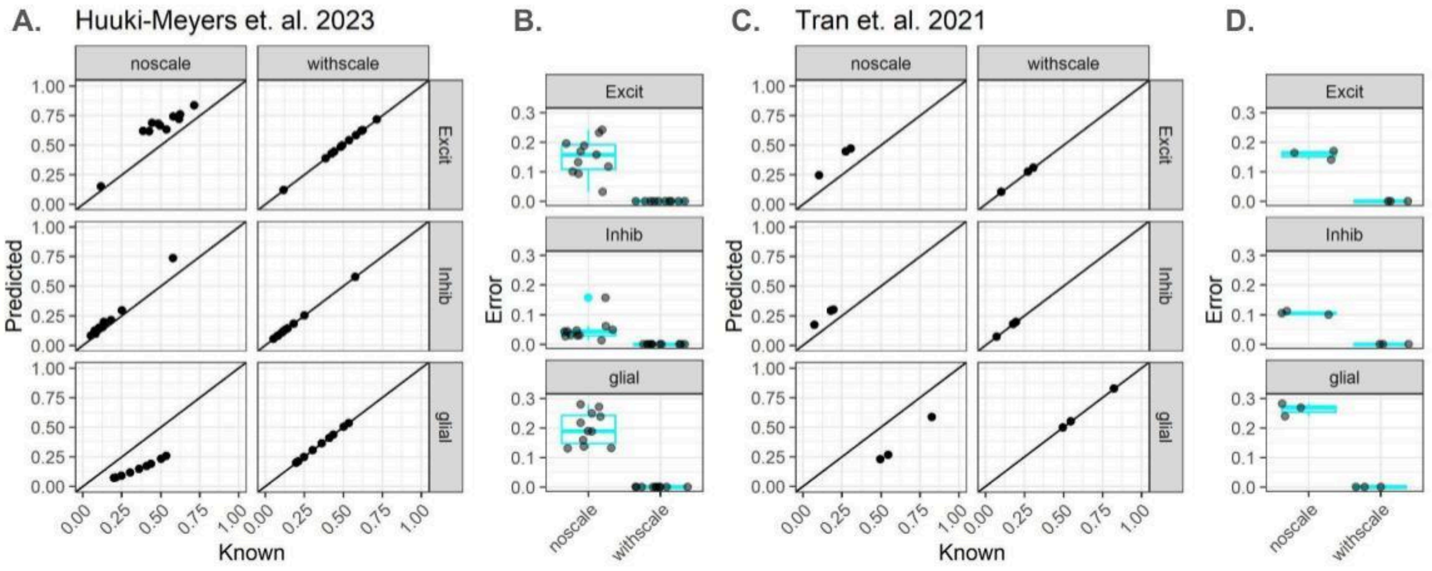

Figure S2 | Pseudobulk simulation results from DLPFC datasets with *k*=3 cell types. (**A-B**) Pseudobulk results from Huuki-Myers et al. (46). (**A**) Scatterplots of estimated the cell composition using *k*=3 (excitatory neuron, inhibitory neuron, and glia) using *NNLS* without (left) and with (right) scaling for differences in cell sizes; the known cell composition is on the *x*-axis and the estimated cell composition is on the *y*-axis. The figure is faceted by cell types (neuron and glia) along the rows. (**B**) Boxplots of the error (difference between the known and predicted cell composition) for the *N*=12 pseudobulk samples. (**C-D**) Pseudobulk results from Tran et al. (2021) (49). (**C**) Scatterplots of the cell composition using *k*=3 (excitatory neuron, inhibitory neuron, and glia) using *NNLS* without (left) and with (right) scaling for differences in cell sizes where the known cell composition is on the *x*-axis and the estimated cell composition is on the *y*-axis. The figure is faceted by cell types (neuron and glia) along the rows. (**D**) Boxplots of the error (difference between the known and predicted cell composition) for the *N*=12 pseudobulk samples. Diagonal lines indicate y = x and no error.

bioRxiv preprint doi: https://doi.org/10.1101/2024.04.04.588105; this version posted April 6, 2024. The copyright holder for this preprint (which was not certified by peer review) is the author/funder, who has granted bioRxiv a license to display the preprint in perpetuity. It is made available under aCC-BY 4.0 International license.

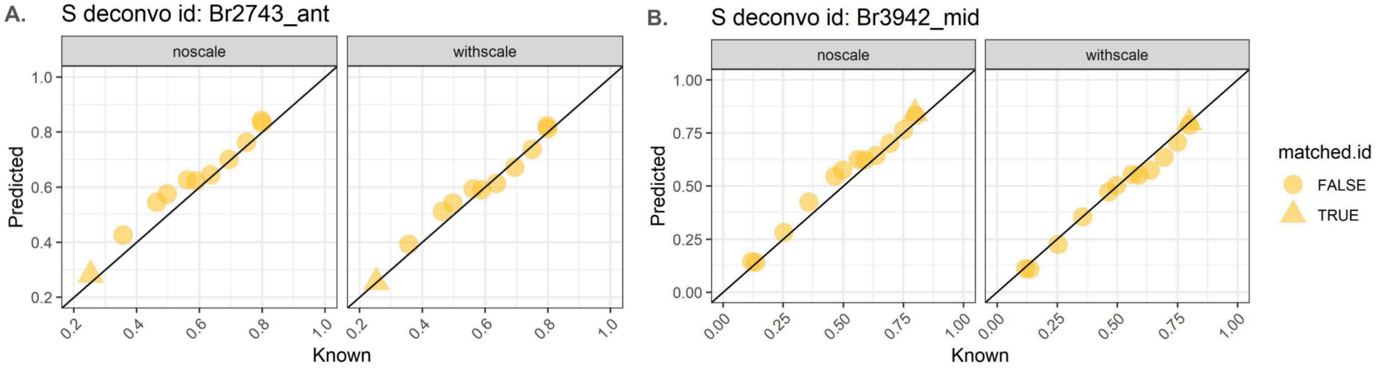

Figure S3 | Impact of randomly shuffling RNAScope cell scale factors in pseudobulk simulations. (**A**) Scatterplots of either (left panel) with or (right panel) without adjusting by cell sizes from sample with low neuron proportions (plot title, Br2743_ant). (**B**) Scatterplots of either (left panel) with or (right panel) without adjusting by cell sizes from sample with high neuron proportions (plot title, Br3942_mid, **Table S7**). Points correspond to if the cell sizes were matched (triangle) or unmatched (circle), where references and cell scale factor arrays were calculated from DLPFC dataset (47). Diagonal lines indicate y = x and no error.

bioRxiv preprint doi: https://doi.org/10.1101/2024.04.04.588105; this version posted April 6, 2024. The copyright holder for this preprint (which was not certified by peer review) is the author/funder, who has granted bioRxiv a license to display the preprint in perpetuity. It is made available under aCC-BY 4.0 International license.

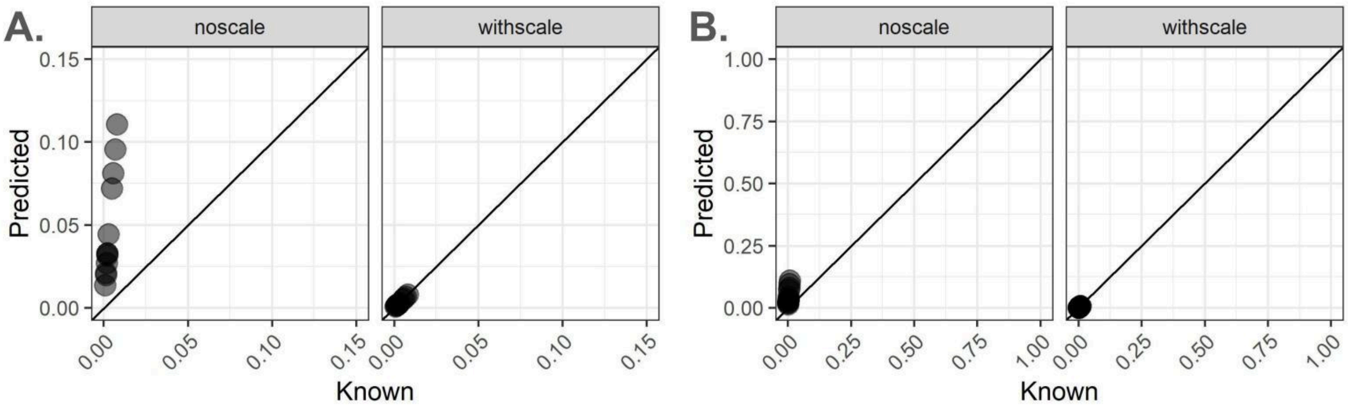

Figure S4 | Deconvolution results before and after rescaling in an independent PBMC experiment. Results are shown for *N*=12 samples from (15) with known proportions from flow cytometry (**Methods**) at two zoom levels, either (**A**) axis maximum = 0.15 or (**B**) axis maximum = 1. Scatterplots show the (x-axis) known flow cytometry proportions versus the (y-axis) predicted proportions of Plasmablasts either (right panel) before or (left panel) after rescaling on cell size scale factors. Diagonal lines indicate y = x and no error.

bioRxiv preprint doi: https://doi.org/10.1101/2024.04.04.588105; this version posted April 6, 2024. The copyright holder for this preprint (which was not certified by peer review) is the author/funder, who has granted bioRxiv a license to display the preprint in perpetuity. It is made available under aCC-BY 4.0 International license.

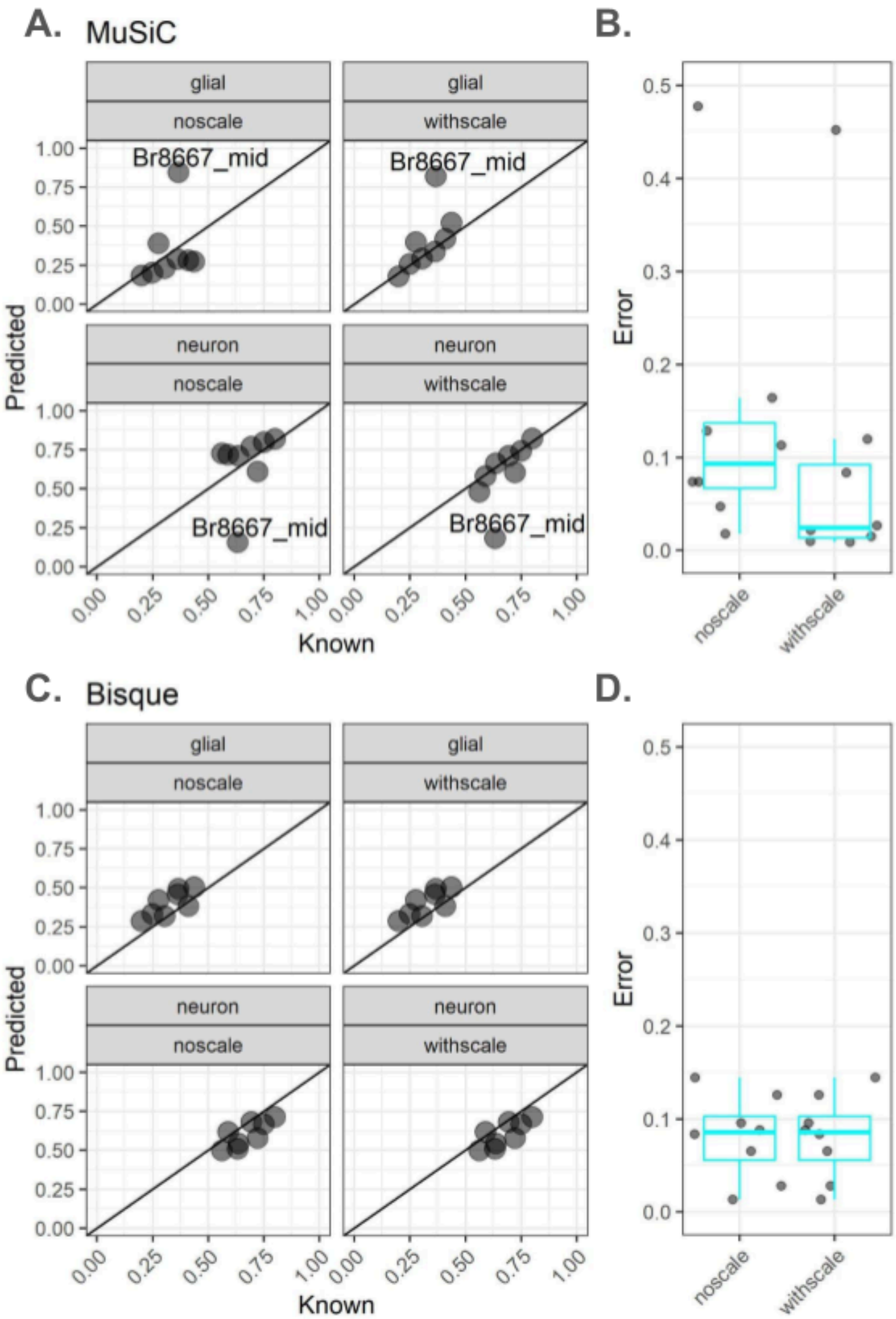

Figure S5 | Results of neuron predictions across deconvolution algorithms in experimental DLPFC RNA-seq samples from (46). (**A**) Scatterplots show results from *MuSiC* in (points) experimental DLPFC bulk RNA-seq samples (top row) glial and (bottom row) neurons either (left column, "noscale") without scaling or (right column, "withscale") with scaling, with text label indicating outlying sample. Diagonal lines indicate y = x and no error. (**B**) Jittered points and quantile boxplots of (y-axis) errors by (x-axis) scaling. (**C**) Scatterplots show results from *Bisque* in (points) real bulk RNA-seq samples (top row) glial and (bottom row) neurons either (left column, "noscale") without scaling or (right column, "withscale") with scaling. Diagonal lines indicate y = x and no error. (**D**) Jittered points and quantile boxplots of (y-axis) errors by (x-axis) scaling.

bioRxiv preprint doi: https://doi.org/10.1101/2024.04.04.588105; this version posted April 6, 2024. The copyright holder for this preprint (which was not certified by peer review) is the author/funder, who has granted bioRxiv a license to display the preprint in perpetuity. It is made available under aCC-BY 4.0 International license.

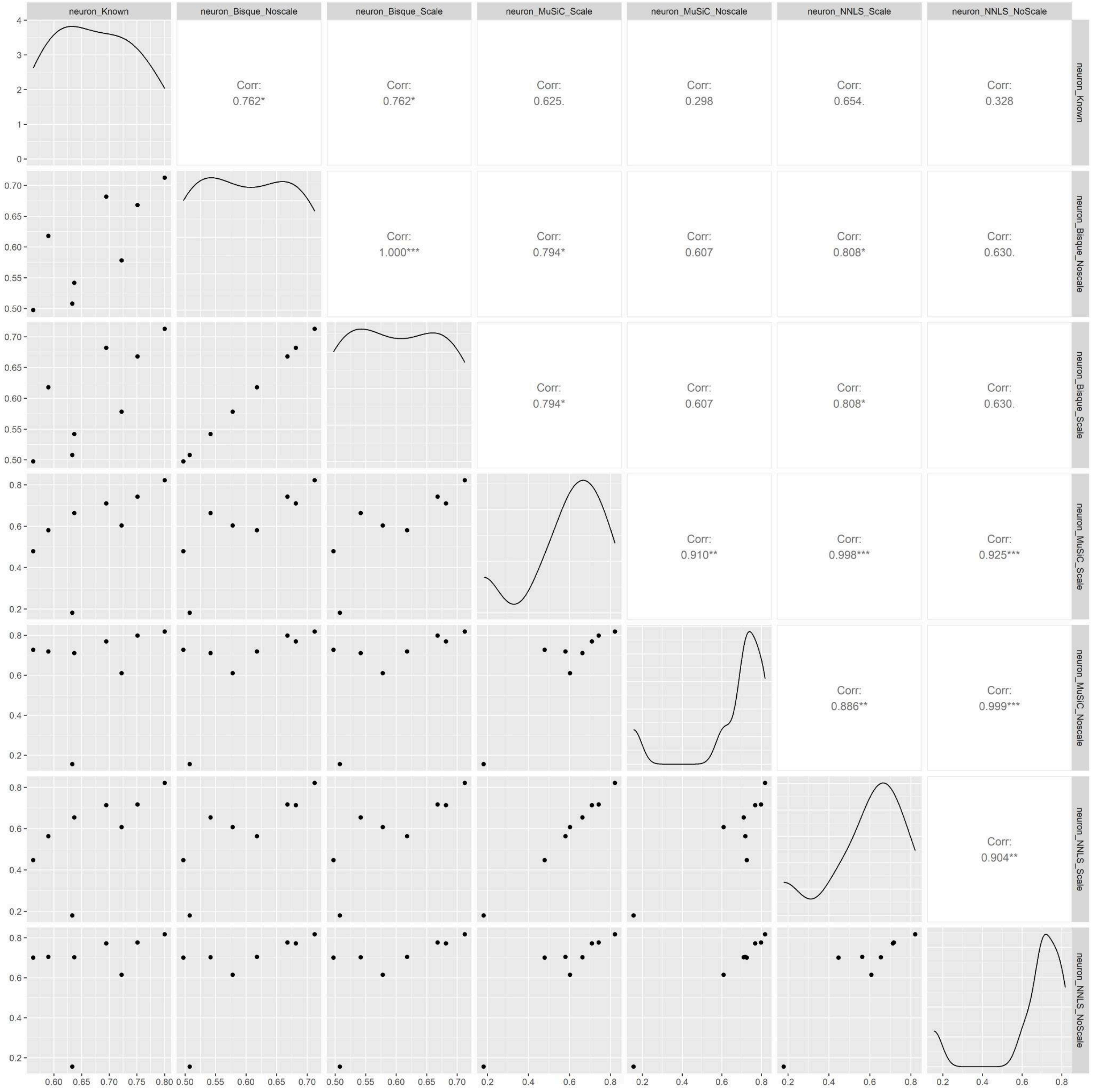

Figure S6 | Correlations of predicted and known neurons in experimental DLPFC bulk RNA-seq, from (47), for *NNLS*, *MuSiC*, and *Bisque*. Pairs plots generated using `GGally` of known and predicted neuron proportions from multiple algorithms in real bulk RNA-seq samples from multiple preparation conditions, for neuron. Row and column labels indicate the cell type, algorithm (either “nnls” for *NNLS* (30), “music” for *MuSiC* (55), or “bisque” for *Bisque* (9), or known), and condition (either scale or noscale). Text panels contain the Pearson R correlation magnitude, with asterisks indicating significance (none : 0.10 <= p; . : 0.05 < p < 0.10; * : 0.01 < p < 0.05; ** : $1.0*10^{-3}$ < p < 0.01; *** : p < $1.0*10^{-3}$).

bioRxiv preprint doi: https://doi.org/10.1101/2024.04.04.588105; this version posted April 6, 2024. The copyright holder for this preprint (which was not certified by peer review) is the author/funder, who has granted bioRxiv a license to display the preprint in perpetuity. It is made available under aCC-BY 4.0 International license.

A. DLPFC (Huuki-Meyers et. al. 2023)

donor_id	donor_index	pseudobulk_index	z	s	p_known
Br2720_mid	1	1			
Br6471_mid	2	2			
Br8492_post	3	3			
Br2743_ant	4	4			
Br8492_mid	5	5			
Br3942_ant	6	6			
Br6423_post	7	7			
Br6423_ant	8	8			
Br3942_mid	9	9			
Br8325_ant	10	10			
Br8325_mid	11	11			
Br2743_mid	12	12			
Br6432_ant	13	13			
Br6522_mid	14	14			
Br6522_post	15	15			
Br8667_mid	16	16			
Br8667_ant	17	17			

B. PBMC (Monaco et. al. 2019)

donor_id	donor_index	pseudobulk_index	z	s	p_known
NA	1	NA			
NA	2	NA			
NA	3	NA			
NA	4	NA			
CYFZ	5	1			
FY2H	6	2			
FLWA	7	3			
453W	8	4			
684C	9	5			
CZJE	10	6			
925L	11	7			
9JD4	12	8			
G4YW	13	9			
4DUY	14	10			
36TS	15	11			
CR3L	16	12			

Available		Unavailable	

Figure S7 | Availability of samples by cohort for pseudobulk experiments in two cohorts and two tissues. (**A**) Availability of samples for pseudobulk experiments in Huuki-Myers et al. (46), with columns indicating (left to right) donor identifier, donor index, pseudobulk index, Z, S, and P_known. (**B**) Availability of samples for pseudobulk experiments in Monaco et. al. 2019 (50), with columns indicating (left to right) donor identifier, donor index, pseudobulk index, Z, S, and P_known. Details about pseudobulk data types provided in **Methods**. Cell colors for Z, S, and P_known indicate data was either (blue) available or (yellow) unavailable for analysis.

bioRxiv preprint doi: https://doi.org/10.1101/2024.04.04.588105; this version posted April 6, 2024. The copyright holder for this preprint (which was not certified by peer review) is the author/funder, who has granted bioRxiv a license to display the preprint in perpetuity. It is made available under aCC-BY 4.0 International license.

Supplemental tables

Table S1 | Tissue block-level summary of Huuki-Myers et al. (2023) (46) DLPFC *N*=17 snRNA-seq libraries generated from tissue blocks obtained from 10 adult neurotypical donors across three position across the dorsolateral prefrontal cortex (DLPFC). These snRNA-seq samples were used in the *in silico* pseudobulk experiments in this paper. Columns summarize information about the snRNA-seq libraries including the number of donors the tissue blocks originated from, the percent of samples that are female, DLPFC anterior to posterior position (position), percent of DLPFC samples stratified by position (posterior, middle, anterior), and the number of nuclei per sample (mean, median, sd, total).

Table S2 | Cell type-level summary of Huuki-Myers et al. (2023) (46) DLPFC *N*=17 snRNA-seq libraries generated from tissue blocks obtained from 10 adult neurotypical donors across three regions of the dorsolateral prefrontal cortex (DLPFC). Summaries are aggregated to *k*=2 (first two rows) or *k*=3 (last two rows) cell types. Columns include *k* dimensions (total cell types), cell type label, nuclei summaries by tissue block (median, mean, sd), proportion summaries (median, mean, sd), library summaries (mean, sd), and pseudobulk cell type scale factor.

Table S3 | Results from estimating the cell composition using the pseudobulk tissue samples from three different sources of snRNA-seq. Three sources of data include snRNA-seq libraries from Huuki-Myers et al. 2023, Tran et al. 2021, and Monaco et al. 2019. Columns include condition ("withscale" if cell size adjustment was used, "noscale" if no adjustment was used, "all" if both adjustment conditions were combined, and an algorithm name where *NNLS* was used if no algorithm name was specified), root mean squared error (RMSE), dataset (represents the source of where the data came from), k_total (total cell types considered in pseudobulk experiment), k_rmse (total cell types used in RMSE calculation), cell type labels in the calculation (separated by ";"), and experiment (type of experiment performed, either "pseudobulk" where pseudobulks were tested, "shuffle" where cell sizes were shuffled across references, or "bulk" where real bulk RNA-seq samples were used).

Table S4 | Sample-level summary of Tran et al. (2021) (49) DLPFC *N*=3 snRNA-seq libraries generated from all posterior tissue blocks in DLPFC obtained from 3 adult neurotypical donors. These snRNA-seq samples were used in the *in silico* pseudobulk experiments in this paper. Columns summarize information about the snRNA-seq libraries including the number of donors the tissue blocks originated from, the percent of samples that are female, DLPFC orientation (region), percent by DLPFC samples stratified by subregion (posterior, middle, anterior), and the number of nuclei per sample (mean, median, sd, total).

Table S5 | Cell type-level summary of Tran et al. (2021) (49) DLPFC *N*=3 snRNA-seq libraries generated from 3 adult neurotypical donors across the posterior region of the dorsolateral prefrontal cortex (DLPFC). Summaries are aggregated to *k*=2 (first two rows) or *k*=3 (last two rows) cell types. Columns include *k* dimensions (total cell types), cell type label, nuclei summaries by tissue block (median, mean, sd), proportion summaries (median, mean, sd), library summaries (mean, sd), and pseudobulk cell type scale factor.

Table S6 | Summary of cell sizes estimated from Huuki-Myers et al. 2024 (26). Columns include sample id, DLPFC region, subject identifier (corresponding to first part of sample id), library-based cell size scale factors and their ratios from snRNA-seq ("sn") and RNAScope ("rn") for neuron and glial.

Table S7 | Results of shuffle analyses of Huuki-Myers et al. (2023) (46) DLPFC *N*=13 snRNA-seq libraries for neuron and glial cell types. (left to right) Columns include known proportions, predictions, cell type label, sample id (id of sample with cell sizes for shuffle experiment), error, index sample id (id of source for pseudobulk), shuffle term (location in deconvolution function), and sizes of neuron and glial cells (from

bioRxiv preprint doi: https://doi.org/10.1101/2024.04.04.588105; this version posted April 6, 2024. The copyright holder for this preprint (which was not certified by peer review) is the author/funder, who has granted bioRxiv a license to display the preprint in perpetuity. It is made available under aCC-BY 4.0 International license.

pseudobulk source or index sample id), and panel indicating the corresponding to **Figure S3** panels. Rows 2 and 3 correspond to the concordant sample (sample id equals index sample id column, Br3942_mid) featured in shuffle experiment in **Figure S3B**, and rows 21 and 22 correspond to concordant sample in **Figure S3A** (Br2743_ant).

Table S8 | Platform-level data summaries of Overview of paired bulk-RNA-seq, snRNA-seq and smFISH datasets from adult neurotypical postmortem human DLPFC tissue blocks. Columns include platform name and sample preparation, and quantities of samples and sample sources (“donors”).
